# 1-(2,2-Di­phenyl­ethen-1-yl)tropylium perchlorate

**DOI:** 10.1107/S2414314626004645

**Published:** 2026-05-15

**Authors:** Dieter Schollmeyer, Heiner Detert

**Affiliations:** a University of Mainz, Department of Chemistry, Duesbergweg 10-14, 55099 Mainz, Germany; Goethe-Universität Frankfurt, Germany

**Keywords:** crystal structure, tropylium, conjugated π-system

## Abstract

In the title salt, C_21_H_17_^+^·ClO_4_^−^, two hydrogen bonds from the tropylium moiety and the vinyl group connect the cation with oxygen atoms of the perchlorate anion. The perchlorate anion is surrounded by three tropylium cations. The tropylium rings of the cations, which are related *via* a C_2_ axis, are mostly parallel with a short distance between the centroids.

## Abstract 2

In the title salt, two hydrogen bonds from the tropylium moiety and the vinyl group connect the cation with oxygen atoms of the perchlorate anion. The perchlorate anion is surrounded by three tropylium cations. The tropylium rings of the cations, which are related *via* a C_2_ axis, are mostly parallel with a short distance between the centroids.

## Graphical abstract

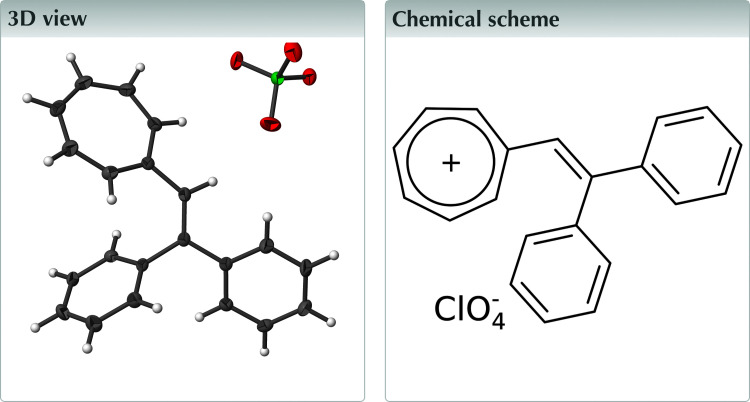

## Structure description

**Figure 1:**
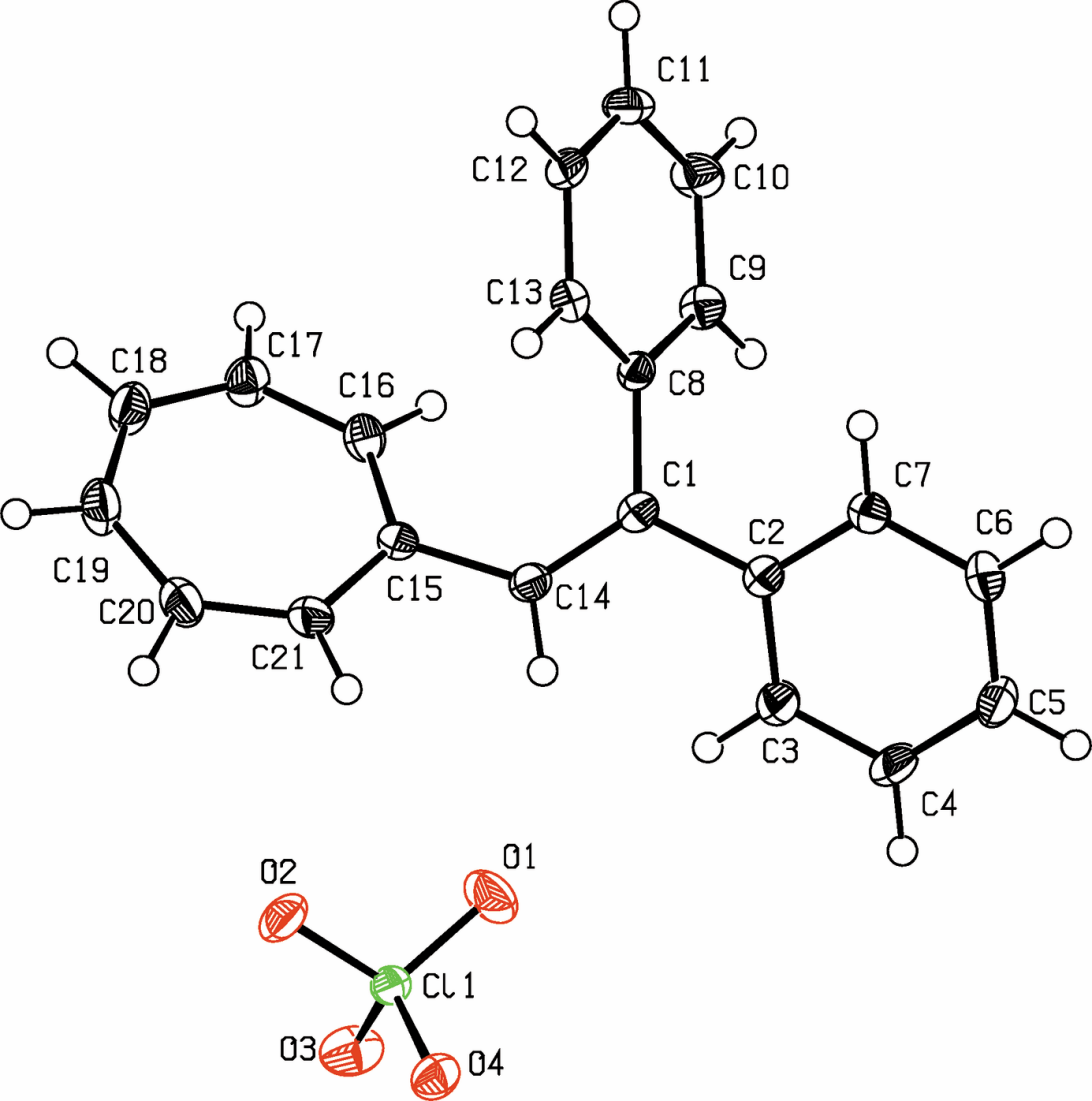
View of the title compound. Displacement ellipsoids are drawn at the 50% probability level.

The title compound, C_21_H_17_^+^·ClO_4_^−^ (Fig. 1 ▸), was reported by Jutz & Voitenleitner (1964 ▸) as part of a series of polymethine dyes. An analogous system with fluorene has been tested as starting material for an all-carbon stable carbene (Alcarazo *et al.*, 2010 ▸). Three essentially planar rings are attached to a vinyl group. This central unit is almost planar [torsion angles: C2—C1—C14—C15 = −177.6 (2)° and C8—C1—C14—C15 = 7.8 (4)°)]. All rings are essentially planar but significant torsion angles between rings and the vinyl group are noted. The tropylium system C15–C21 and the ethene moiety include a C21—C15—C14—C1 torsion angle of −152.8 (2)° while the torsion angle between the *trans*-phenyl ring C2–C7 and the ethene moiety is to 27.4 (3)° (C3—C2—C1—C14) and the torsion angle between the ethene group and the *cis*-phenyl ring is −128.9 (2)° (C9—C8—C1—C14). The least-squares planes of the two phenyl rings subtend a dihedral angle of 73.43 (8)°, the angles between the tropylium and phenyl rings are smaller: 60.35 (8)° for tropylium and *trans*-phen­yl and only only 50.76 (8)° for tropylium and *cis*-phenyl.

**Figure 2:**
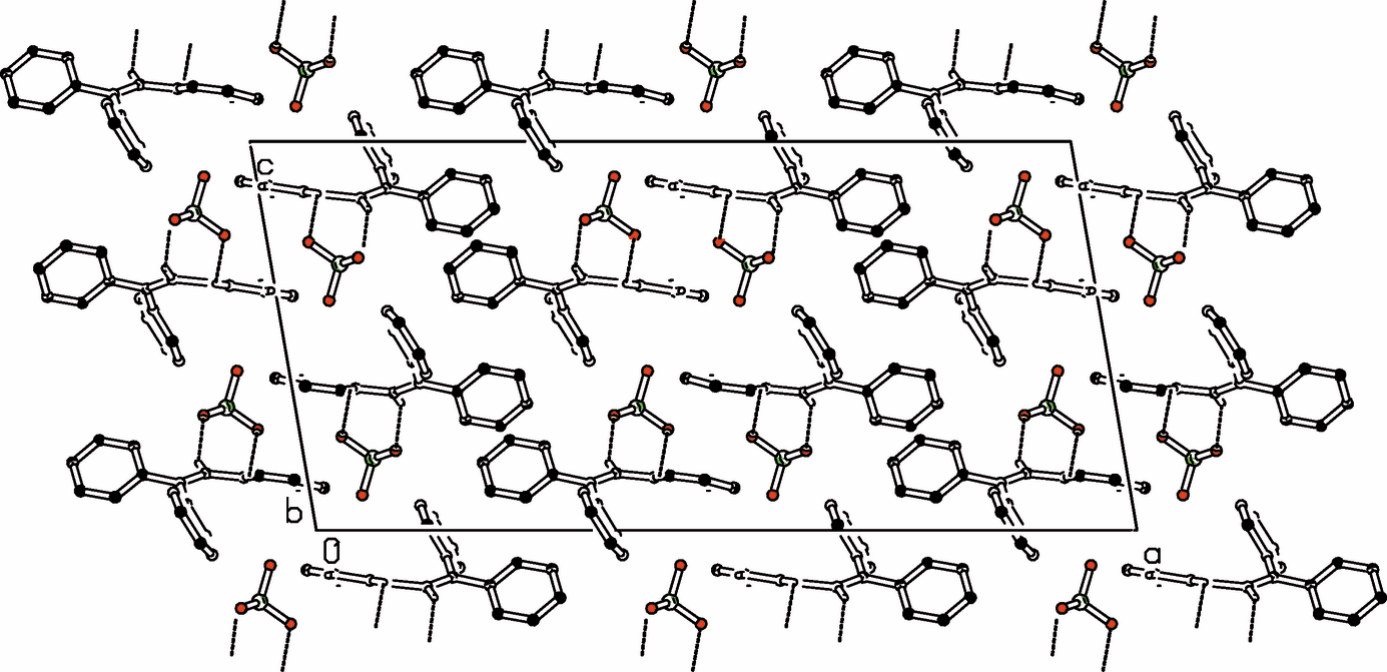
Part of the packing diagram. Hydrogen bonds are shown as dashed lines. View along the *b*-axis direction. Only hydrogen atoms involved in hydrogen bonds are shown for clarity.

Hydrogen bonds connect the perchlorate ion to the cation: one to the vinyl group [C14—H14⋯O1: 3.347 (3) Å with H14⋯O1 = 2.49 Å and a bond angle of 151°], the other to tropylium: [C21—H21⋯O2 = 3.173 (3) Å with H21⋯O2 = 2.55 (3) Å and an angle of 123 (2)°] (Table 1 ▸, Fig. 2 ▸). Eight ion pairs fill the monoclinic unit cell. The perchlorate ion is closely connected *via* hydrogen bonds to one cation. In the *ac* plane, the perchlorate ion is surrounded by three tropylium rings. Small distances of 3.8756 (14) Å between tropylium centroids, a small dihedral angle of 6.98 (10)° and a small slippage (0.451 Å) indicate a significant π–π inter­action. These units are connected *via C*_2_ symmetry.

## Synthesis and crystallization

The title compound was prepared according to Jutz & Voitenleitner (1964 ▸) in a Grignard reaction of 2,2-di­phenyl­vinyl bromide and meth­oxy­cyclo­hepta­triene followed by deprotonation with tritylium perchlorate. Dark red–violet crystals, m*.*p. 457 K. ^1^H-NMR (CD_2_Cl_2_, 400 MHz): 7.26 (*dm*, 2 H, *J* = 7 Hz), 7.31–7.53 (*m*, 6 H), 7.54 (*s*, 1 H), 7.55 (*m*, 2H), 8.54–8.61 (*m*, 2 H), 8.64 (*d*, *J* = 9 Hz, 2 H), 8.68–8,73 (*m*, 2 H). ^13^C-NMR (CD_2_Cl_2_, 100 MHz): 128.2 (CH), 129.22 (2 CH), 129.84 (2 CH), 130.39 (2 CH), 131.16 (2 CH), 131.23 (CH), 131.93 (CH), 136.58 (Cq), 140.71 (Cq), 151.08 (2 CH), 151.79 (2 CH), 153.40 (2 CH), 161.29 (Cq), 167.89 (Cq).

## Refinement

Crystal data, data collection and structure refinement details are summarized in Table 2 ▸.

## Supplementary Material

Crystal structure: contains datablock(s) I, global. DOI: 10.1107/S2414314626004645/bt4198sup1.cif

Structure factors: contains datablock(s) I. DOI: 10.1107/S2414314626004645/bt4198Isup2.hkl

Supporting information file. DOI: 10.1107/S2414314626004645/bt4198Isup3.cml

CCDC reference: 2551594

Additional supporting information:  crystallographic information; 3D view; checkCIF report

## Figures and Tables

**Table 1:** Hydrogen-bond geometry (Å, °)

*D*—H⋯*A*	*D*—H	H⋯*A*	*D*⋯*A*	*D*—H⋯*A*
C14—H14⋯O1	0.95	2.49	3.347 (3)	151
C21—H21⋯O2	0.95 (3)	2.55 (3)	3.173 (3)	123 (2)

**Table 2:** Experimental details

Crystal data	
Chemical formula	C_21_H_17_^+^·ClO_4_^−^
*M*_r_	368.80
Crystal system, space group	Monoclinic, *C*2/*c*
Temperature (K)	120
*a*, *b*, *c* (Å)	31.1356 (11), 7.7899 (2), 14.9686 (6)
β (°)	99.678 (3)
*V* (Å^3^)	3578.9 (2)
*Z*	8
Radiation type	Mo *K*α
μ (mm^−1^)	0.24
Crystal size (mm)	0.98 × 0.29 × 0.10

Data collection	
Diffractometer	Stoe *IPDS* 2T
Absorption correction	Integration
*T*_min_, *T*_max_	0.901, 0.981
No. of measured, independent and observed [*I* > 2σ(*I*)] reflections	9146, 4227, 3203
*R*_int_	0.041
(sin θ/λ)_max_ (Å^−1^)	0.658

Refinement	
*R*[*F*^2^ > 2σ(*F*^2^)], *wR*(*F*^2^), *S*	0.054, 0.135, 1.06
No. of reflections	4227
No. of parameters	253
H-atom treatment	H atoms treated by a mixture of independent and constrained refinement
Δρ_max_, Δρ_min_ (e Å^−3^)	0.37, −0.43

Computer programs: *X-AREA WinXpose*, *Recipe* and *Integrate*(Stoe & Cie, 2020 ▸), *SHELXT2014* (Sheldrick, 2015*a* ▸), *SHELXL2019/2* (Sheldrick, 2015*b* ▸) and *PLATON* (Spek, 2009 ▸).

## full crystallographic data

### 1-(2,2-Diphenylethen-1-yl)cyclohepta-2,4,6-trien-1-ylium perchlorate Crystal data

**Table:**

C_21_H_17_^+^·ClO_4_^−^	*D*_x_ = 1.369 Mg m^−^^3^
*M**_r_* = 368.80	Melting point: 457 K
Monoclinic, *C*2/*c*	Mo *K*α radiation, λ = 0.71073 Å
*a* = 31.1356 (11) Å	Cell parameters from 8512 reflections
*b* = 7.7899 (2) Å	θ = 2.7–28.4°
*c* = 14.9686 (6) Å	µ = 0.24 mm^−^^1^
β = 99.678 (3)°	*T* = 120 K
*V* = 3578.9 (2) Å^3^	Plate, brown
*Z* = 8	0.98 × 0.29 × 0.10 mm
*F*(000) = 1536	

### 1-(2,2-Diphenylethen-1-yl)cyclohepta-2,4,6-trien-1-ylium perchlorate Data collection

**Table:**

Stoe IPDS 2T diffractometer	3203 reflections with *I* > 2σ(*I*)
Detector resolution: 6.67 pixels mm^-1^	*R*_int_ = 0.041
rotation method, ω scans	θ_max_ = 27.9°, θ_min_ = 2.7°
Absorption correction: integration	*h* = −40→40
*T*_min_ = 0.901, *T*_max_ = 0.981	*k* = −10→10
9146 measured reflections	*l* = −19→13
4227 independent reflections	

### 1-(2,2-Diphenylethen-1-yl)cyclohepta-2,4,6-trien-1-ylium perchlorate Refinement

**Table:**

Refinement on *F*^2^	Primary atom site location: dual
Least-squares matrix: full	Hydrogen site location: mixed
*R*[*F*^2^ > 2σ(*F*^2^)] = 0.054	H atoms treated by a mixture of independent and constrained refinement
*wR*(*F*^2^) = 0.135	*w* = 1/[σ^2^(*F*_o_^2^) + (0.051*P*)^2^ + 8.3409*P*] where *P* = (*F*_o_^2^ + 2*F*_c_^2^)/3
*S* = 1.06	(Δ/σ)_max_ = 0.001
4227 reflections	Δρ_max_ = 0.37 e Å^−^^3^
253 parameters	Δρ_min_ = −0.42 e Å^−^^3^
0 restraints	

### 1-(2,2-Diphenylethen-1-yl)cyclohepta-2,4,6-trien-1-ylium perchlorate Special details

**Table:**

Geometry. All esds (except the esd in the dihedral angle between two l.s. planes) are estimated using the full covariance matrix. The cell esds are taken into account individually in the estimation of esds in distances, angles and torsion angles; correlations between esds in cell parameters are only used when they are defined by crystal symmetry. An approximate (isotropic) treatment of cell esds is used for estimating esds involving l.s. planes.
Refinement. Hydrogen atoms attached to phenyl and ethene carbon atoms were placed at calculated positions and were refined in the riding-model approximation with C–H = 0.99 Å, and with *U*_iso_(H) = 1.2 *U*_eq_(C). The coordinates of the hydrogen atoms of the tropylium cation were refined with *U*_iso_(H) = 1.2 *U*_eq_(C).

### 1-(2,2-Diphenylethen-1-yl)cyclohepta-2,4,6-trien-1-ylium perchlorate Fractional atomic coordinates and isotropic or equivalent isotropic displacement parameters (Å^2^)

**Table:**

	*x*	*y*	*z*	*U*_iso_*/*U*_eq_	
Cl1	0.41609 (2)	0.29334 (7)	0.81544 (4)	0.01892 (14)	
O1	0.38676 (6)	0.4359 (2)	0.79513 (14)	0.0363 (5)	
O2	0.44931 (6)	0.3007 (2)	0.75909 (13)	0.0303 (4)	
O3	0.43658 (6)	0.3005 (2)	0.90919 (12)	0.0332 (4)	
O4	0.39243 (5)	0.1345 (2)	0.79900 (12)	0.0257 (4)	
C1	0.34337 (7)	0.8688 (3)	0.62122 (15)	0.0177 (4)	
C2	0.29973 (7)	0.8240 (3)	0.64183 (15)	0.0180 (4)	
C3	0.29527 (8)	0.7190 (3)	0.71512 (17)	0.0280 (5)	
H3	0.320546	0.681002	0.754824	0.034*	
C4	0.25442 (8)	0.6692 (4)	0.73085 (19)	0.0323 (6)	
H4	0.251951	0.598505	0.781455	0.039*	
C5	0.21727 (7)	0.7218 (3)	0.67336 (17)	0.0267 (5)	
H5	0.189381	0.685822	0.683784	0.032*	
C6	0.22093 (7)	0.8267 (3)	0.60080 (16)	0.0224 (5)	
H6	0.195461	0.863167	0.561233	0.027*	
C7	0.26197 (7)	0.8797 (3)	0.58518 (15)	0.0192 (4)	
H7	0.264178	0.953945	0.535816	0.023*	
C8	0.34711 (7)	1.0256 (3)	0.56708 (15)	0.0173 (4)	
C9	0.33371 (7)	1.1853 (3)	0.59630 (16)	0.0218 (5)	
H9	0.321355	1.192816	0.650043	0.026*	
C10	0.33857 (8)	1.3316 (3)	0.54661 (18)	0.0268 (5)	
H10	0.329943	1.439723	0.567043	0.032*	
C11	0.35600 (8)	1.3220 (3)	0.46682 (18)	0.0268 (5)	
H11	0.359674	1.423210	0.433474	0.032*	
C12	0.36792 (7)	1.1638 (3)	0.43660 (16)	0.0226 (5)	
H12	0.379058	1.156112	0.381405	0.027*	
C13	0.36371 (7)	1.0165 (3)	0.48636 (15)	0.0187 (4)	
H13	0.372196	0.908645	0.465320	0.022*	
C14	0.37713 (7)	0.7594 (3)	0.64594 (16)	0.0194 (4)	
H14	0.370215	0.655682	0.673732	0.023*	
C15	0.42274 (7)	0.7792 (3)	0.63569 (15)	0.0190 (4)	
C16	0.44191 (7)	0.9440 (3)	0.64043 (16)	0.0214 (5)	
H16	0.4243 (9)	1.034 (4)	0.6558 (19)	0.026*	
C17	0.48350 (7)	0.9909 (3)	0.62940 (18)	0.0251 (5)	
H17	0.4887 (9)	1.109 (4)	0.639 (2)	0.030*	
C18	0.51770 (8)	0.8898 (3)	0.60906 (19)	0.0277 (5)	
H18	0.5431 (9)	0.951 (4)	0.600 (2)	0.033*	
C19	0.51942 (8)	0.7143 (3)	0.60262 (19)	0.0291 (5)	
H19	0.5455 (10)	0.667 (4)	0.592 (2)	0.035*	
C20	0.48716 (8)	0.5961 (3)	0.6146 (2)	0.0304 (6)	
H20	0.4949 (9)	0.477 (4)	0.616 (2)	0.036*	
C21	0.44509 (8)	0.6241 (3)	0.62903 (18)	0.0260 (5)	
H21	0.4286 (9)	0.523 (4)	0.634 (2)	0.031*	

### 1-(2,2-Diphenylethen-1-yl)cyclohepta-2,4,6-trien-1-ylium perchlorate Atomic displacement parameters (Å^2^)

**Table:**

	*U*^11^	*U*^22^	*U*^33^	*U*^12^	*U*^13^	*U*^23^
Cl1	0.0196 (2)	0.0177 (3)	0.0203 (3)	0.00058 (19)	0.00565 (19)	0.0014 (2)
O1	0.0407 (10)	0.0284 (10)	0.0420 (12)	0.0180 (8)	0.0132 (9)	0.0049 (8)
O2	0.0292 (9)	0.0293 (9)	0.0372 (11)	−0.0029 (7)	0.0196 (8)	0.0024 (8)
O3	0.0414 (10)	0.0349 (10)	0.0212 (9)	−0.0102 (8)	−0.0009 (8)	0.0008 (8)
O4	0.0247 (8)	0.0246 (9)	0.0279 (9)	−0.0061 (7)	0.0045 (7)	0.0000 (7)
C1	0.0195 (10)	0.0173 (10)	0.0171 (11)	−0.0019 (8)	0.0054 (8)	−0.0023 (8)
C2	0.0194 (10)	0.0169 (10)	0.0185 (11)	−0.0006 (8)	0.0053 (8)	−0.0025 (8)
C3	0.0203 (11)	0.0370 (14)	0.0267 (13)	0.0011 (10)	0.0044 (9)	0.0105 (11)
C4	0.0257 (12)	0.0434 (15)	0.0296 (15)	−0.0026 (11)	0.0102 (10)	0.0168 (12)
C5	0.0187 (10)	0.0330 (13)	0.0297 (13)	−0.0027 (10)	0.0083 (9)	0.0007 (10)
C6	0.0172 (10)	0.0270 (12)	0.0221 (12)	0.0027 (9)	0.0005 (9)	−0.0027 (9)
C7	0.0218 (10)	0.0185 (10)	0.0177 (11)	0.0020 (8)	0.0047 (8)	−0.0008 (8)
C8	0.0150 (9)	0.0186 (10)	0.0183 (11)	−0.0020 (8)	0.0027 (8)	0.0008 (8)
C9	0.0243 (11)	0.0220 (11)	0.0196 (11)	0.0001 (9)	0.0055 (9)	−0.0016 (9)
C10	0.0345 (13)	0.0171 (11)	0.0300 (14)	0.0020 (10)	0.0092 (10)	−0.0016 (9)
C11	0.0325 (12)	0.0230 (12)	0.0261 (13)	0.0018 (10)	0.0086 (10)	0.0093 (9)
C12	0.0211 (10)	0.0282 (12)	0.0200 (11)	0.0007 (9)	0.0080 (9)	0.0056 (9)
C13	0.0170 (10)	0.0192 (10)	0.0197 (11)	0.0018 (8)	0.0024 (8)	−0.0007 (8)
C14	0.0203 (10)	0.0181 (10)	0.0204 (11)	−0.0016 (8)	0.0053 (8)	0.0016 (8)
C15	0.0184 (10)	0.0210 (10)	0.0173 (11)	0.0008 (8)	0.0021 (8)	0.0041 (9)
C16	0.0203 (11)	0.0222 (11)	0.0215 (12)	0.0022 (9)	0.0029 (9)	−0.0036 (9)
C17	0.0209 (11)	0.0219 (11)	0.0319 (14)	−0.0041 (9)	0.0027 (9)	−0.0044 (10)
C18	0.0184 (11)	0.0298 (13)	0.0352 (15)	−0.0034 (10)	0.0052 (10)	−0.0017 (11)
C19	0.0197 (11)	0.0286 (13)	0.0395 (15)	0.0045 (10)	0.0067 (10)	−0.0041 (11)
C20	0.0230 (11)	0.0232 (12)	0.0443 (17)	0.0052 (10)	0.0041 (11)	0.0044 (11)
C21	0.0229 (11)	0.0187 (11)	0.0355 (15)	−0.0005 (9)	0.0025 (10)	0.0059 (10)

### 1-(2,2-Diphenylethen-1-yl)cyclohepta-2,4,6-trien-1-ylium perchlorate Geometric parameters (Å, º)

**Table:**

Cl1—O1	1.4372 (17)	C10—C11	1.395 (4)
Cl1—O4	1.4397 (17)	C10—H10	0.9500
Cl1—O3	1.4416 (18)	C11—C12	1.385 (3)
Cl1—O2	1.4417 (17)	C11—H11	0.9500
C1—C14	1.356 (3)	C12—C13	1.386 (3)
C1—C8	1.481 (3)	C12—H12	0.9500
C1—C2	1.485 (3)	C13—H13	0.9500
C2—C3	1.394 (3)	C14—C15	1.462 (3)
C2—C7	1.398 (3)	C14—H14	0.9500
C3—C4	1.387 (3)	C15—C21	1.406 (3)
C3—H3	0.9500	C15—C16	1.412 (3)
C4—C5	1.382 (4)	C16—C17	1.382 (3)
C4—H4	0.9500	C16—H16	0.94 (3)
C5—C6	1.379 (3)	C17—C18	1.399 (3)
C5—H5	0.9500	C17—H17	0.94 (3)
C6—C7	1.399 (3)	C18—C19	1.372 (4)
C6—H6	0.9500	C18—H18	0.95 (3)
C7—H7	0.9500	C19—C20	1.396 (4)
C8—C13	1.394 (3)	C19—H19	0.93 (3)
C8—C9	1.405 (3)	C20—C21	1.380 (3)
C9—C10	1.383 (3)	C20—H20	0.96 (3)
C9—H9	0.9500	C21—H21	0.95 (3)
			
O1—Cl1—O4	109.82 (11)	C11—C10—H10	119.7
O1—Cl1—O3	109.73 (12)	C12—C11—C10	119.4 (2)
O4—Cl1—O3	109.24 (10)	C12—C11—H11	120.3
O1—Cl1—O2	109.77 (11)	C10—C11—H11	120.3
O4—Cl1—O2	109.34 (11)	C11—C12—C13	120.4 (2)
O3—Cl1—O2	108.92 (11)	C11—C12—H12	119.8
C14—C1—C8	122.62 (19)	C13—C12—H12	119.8
C14—C1—C2	119.36 (19)	C12—C13—C8	120.4 (2)
C8—C1—C2	117.81 (18)	C12—C13—H13	119.8
C3—C2—C7	118.3 (2)	C8—C13—H13	119.8
C3—C2—C1	121.1 (2)	C1—C14—C15	128.9 (2)
C7—C2—C1	120.4 (2)	C1—C14—H14	115.6
C4—C3—C2	120.9 (2)	C15—C14—H14	115.6
C4—C3—H3	119.6	C21—C15—C16	125.1 (2)
C2—C3—H3	119.6	C21—C15—C14	114.7 (2)
C5—C4—C3	120.4 (2)	C16—C15—C14	120.0 (2)
C5—C4—H4	119.8	C17—C16—C15	129.1 (2)
C3—C4—H4	119.8	C17—C16—H16	115.4 (16)
C6—C5—C4	119.6 (2)	C15—C16—H16	115.4 (16)
C6—C5—H5	120.2	C16—C17—C18	129.8 (2)
C4—C5—H5	120.2	C16—C17—H17	112.5 (17)
C5—C6—C7	120.3 (2)	C18—C17—H17	117.7 (17)
C5—C6—H6	119.8	C19—C18—C17	128.1 (2)
C7—C6—H6	119.8	C19—C18—H18	116.4 (17)
C2—C7—C6	120.4 (2)	C17—C18—H18	115.5 (17)
C2—C7—H7	119.8	C18—C19—C20	127.6 (2)
C6—C7—H7	119.8	C18—C19—H19	116.7 (19)
C13—C8—C9	119.2 (2)	C20—C19—H19	115.6 (18)
C13—C8—C1	120.5 (2)	C21—C20—C19	129.7 (2)
C9—C8—C1	120.3 (2)	C21—C20—H20	113.0 (17)
C10—C9—C8	119.8 (2)	C19—C20—H20	117.3 (17)
C10—C9—H9	120.1	C20—C21—C15	129.8 (2)
C8—C9—H9	120.1	C20—C21—H21	114.8 (17)
C9—C10—C11	120.7 (2)	C15—C21—H21	115.4 (17)
C9—C10—H10	119.7		
			
C14—C1—C2—C3	27.4 (3)	C9—C10—C11—C12	1.0 (4)
C8—C1—C2—C3	−157.7 (2)	C10—C11—C12—C13	−1.8 (4)
C14—C1—C2—C7	−149.4 (2)	C11—C12—C13—C8	0.5 (3)
C8—C1—C2—C7	25.4 (3)	C9—C8—C13—C12	1.5 (3)
C7—C2—C3—C4	0.8 (4)	C1—C8—C13—C12	−178.8 (2)
C1—C2—C3—C4	−176.1 (2)	C8—C1—C14—C15	7.8 (4)
C2—C3—C4—C5	0.6 (4)	C2—C1—C14—C15	−177.6 (2)
C3—C4—C5—C6	−1.1 (4)	C1—C14—C15—C21	−152.8 (2)
C4—C5—C6—C7	0.1 (4)	C1—C14—C15—C16	32.3 (4)
C3—C2—C7—C6	−1.8 (3)	C21—C15—C16—C17	8.2 (4)
C1—C2—C7—C6	175.2 (2)	C14—C15—C16—C17	−177.4 (2)
C5—C6—C7—C2	1.3 (3)	C15—C16—C17—C18	1.3 (5)
C14—C1—C8—C13	51.5 (3)	C16—C17—C18—C19	−6.2 (5)
C2—C1—C8—C13	−123.2 (2)	C17—C18—C19—C20	0.3 (5)
C14—C1—C8—C9	−128.9 (2)	C18—C19—C20—C21	5.3 (5)
C2—C1—C8—C9	56.4 (3)	C19—C20—C21—C15	0.0 (5)
C13—C8—C9—C10	−2.3 (3)	C16—C15—C21—C20	−8.9 (4)
C1—C8—C9—C10	178.1 (2)	C14—C15—C21—C20	176.5 (3)
C8—C9—C10—C11	1.1 (4)		

### 1-(2,2-Diphenylethen-1-yl)cyclohepta-2,4,6-trien-1-ylium perchlorate Hydrogen-bond geometry (Å, º)

**Table:**

*D*—H···*A*	*D*—H	H···*A*	*D*···*A*	*D*—H···*A*
C14—H14···O1	0.95	2.49	3.347 (3)	151
C21—H21···O2	0.95 (3)	2.55 (3)	3.173 (3)	123 (2)
